# A *de novo* frameshift variant in the candidate *RBM15* in a proband with congenital mirror movements

**DOI:** 10.1016/j.xhgg.2025.100528

**Published:** 2025-10-07

**Authors:** Frederike L. Harms, Fanny Kortüm, Malik Alawi, Martin Staudt, Kerstin Kutsche

**Affiliations:** 1 Institute of Human Genetics, University Medical Center Hamburg-Eppendorf, 20246 Hamburg, Germany; 2 Bioinformatics Core, University Medical Center Hamburg-Eppendorf, 20246 Hamburg, Germany; 3 Center for Pediatric Palliative Care, Dr. von Hauner Children’s Hospital, Ludwig-Maximilian-University, Campus Grosshadern, 81377 Munich, Germany; 4 German Center for Child and Adolescent Health (DZKJ), Partner Site Hamburg, 20246 Hamburg, Germany

**Keywords:** mirror movements, *DCC*, Netrin-1, NOVA1, NOVA2, RBM15, alternative splicing, autosomal dominant, corticospinal tract, minigene

## Abstract

Congenital mirror movements (CMMs) are involuntary movements of one side of the body that mirror intentional movements of the opposite side. *DCC*, *NTN1*, *RAD51*, *ARHGEF7*, and *DNAL4* have been associated with CMMs. Two-thirds of CMM-affected individuals remain without a genetic diagnosis, indicating that variants in additional genes need to be discovered. We report on a 27-year-old female with CMMs of the hands. Trio exome sequencing in the proband and healthy parents did not reveal a likely pathogenic variant in one of the CMM-associated genes but rather a *de novo* heterozygous frameshift variant c.523dup (p.Ser175Lysfs∗8) in the candidate *RBM15*. The variant results in only partial nonsense-mediated mRNA decay of *RBM15* transcripts in the proband’s lymphoblastoid cells. *RBM15* encodes an RNA-binding protein involved in alternative splicing as well as other processes. *Dcc* alternative splicing generates Dcc_long_ and Dcc_short_ isoforms, which are important for commissural axon midline crossing. We tested whether Rbm15 regulates *Dcc* alternative splicing by using an *in vitro* minigene assay. Ectopic expression of Rbm15, similar to the splicing factors Nova1 and Nova2, promotes the production of *Dcc*_*long*_ transcripts. The possible link between Rbm15 and Dcc supports a role for Rbm15 in CMMs.

## Teaser

We report *RBM15* as a potential novel gene associated with congenital mirror movements (CMMs), characterized by involuntary movements on one side of the body that mirror intentional movements on the opposite side. Our functional studies support a possible role for RBM15 in CMMs.

## Introduction

Congenital mirror movements (CMMs) are involuntary movements of one side of the body that mirror voluntary movements of the opposite side. The onset of CMMs is in infancy or early childhood; they persist throughout life and vary in severity. The upper limbs, especially the fingers, are predominantly affected, resulting in difficulty with activities of daily living. CMMs are associated with abnormal ipsilateral corticospinal tract and bilateral activation of the primary motor cortices during movement preparation and execution.^1^^,^^2^^,^^3^ Thus, abnormal ipsilateral corticospinal tract and bilateral activation of the primary motor cortices likely underlie mirror movements.^1^^,^^2^

CMMs can occur in simplex cases or are inherited in an autosomal-dominant fashion with incomplete penetrance in most of the families. It is a genetically heterogeneous disorder and is caused by heterozygous pathogenic variants in *DCC* (MIM: 120470),^4^ *RAD51* (MIM: 179617),^5^ *NTN1* (MIM: 601614),^6^ and *ARHGEF7* (MIM: 605477),^7^ while a homozygous splice-site variant in *DNAL4* has been reported in a single Pakistani family.^8^ Genetic analysis in large cohorts of individuals with CMMs identified *DCC* as the most commonly mutated gene, while pathogenic variants in *RAD51*, *NTN1*, and *ARHGEF7* were rarely found in affected individuals.^9^^,^^10^^,^^11^ Overall, an underlying genetic cause can be found in about one-third of the affected individuals, with a yield of 70% in those with a positive family history. Thus, the high proportion of individuals with CMMs that are genetically unsolved suggests the existence of additional genes associated with CMMs that need to be discovered.^9^ The lack of reports on novel CMM-associated genes for several years and the identification of pathogenic variants in *DNAL4* and *ARHGEF7* in single families suggest a high degree of genetic heterogeneity, with affected simplex individuals and families possibly carrying a pathogenic variant in their private gene.

DCC, NTN1 (Netrin-1), and ARHGEF7 are required for Netrin-1-stimulated commissural axon outgrowth and guidance.^2^^,^^7^ The secreted protein Netrin-1 binds to the DCC receptor,^12^^,^^13^ and ARHGEF7 is important for Netrin-1-induced increase of DCC at the cell surface of commissural neuron growth cones.^7^ The DCC receptor is a transmembrane protein of the immunoglobulin superfamily of cell adhesion molecules.^12^ Alternative splicing of the first 60 bp of *Dcc* exon 17 generates two isoforms that differ in 20 amino acid residues in the linker region between the fourth and fifth fibronectin repeats, referred to as Dcc_long_ and Dcc_short_^14^^,^^15^ The RNA-binding proteins Nova1 and Nova2 regulate alternative splicing of *Dcc*. *Dcc* alternative splicing is perturbed in a *Nova1*/*Nova2* double-knockout mouse that shows commissural axon guidance defects, demonstrating that alternative splicing of *Dcc* exon 17 is important for spinal commissural neuron development.^14^^,^^16^

Here, we report on a female with CMMs and no pathogenic variant in any of the known genes associated with CMMs, such as *DCC*, *RAD51*, *NTN1*, and *ARHGEF7*. Trio exome sequencing in the proband and parents identified a *de novo* frameshift variant in the candidate gene *RBM15* (MIM: 606077) that likely is a loss-of-function (LoF) allele. Our *in vitro* studies revealed a role for the RNA-binding protein Rbm15 in alternative splicing of *Dcc* by promoting the expression of *Dcc*_*long*_ transcripts, similar to Nova1 and Nova2 splicing factors. Our data suggest that Rbm15 may be involved in regulating the production of the functionally distinct Dcc_long_ and Dcc_short_ isoforms during commissural neuron development.

## Material and methods

### Study approval

The proband and parents provided written informed consent for participation in the study, clinical data and specimen collection, genetic analysis, and publication of relevant findings under a protocol approved by the ethics committee of the Hamburg Medical Chamber (PV7038-4438-BO-ff; Hamburg, Germany).

Detailed methodologies are given in the supplemental information.

## Results

**Figure 1:**
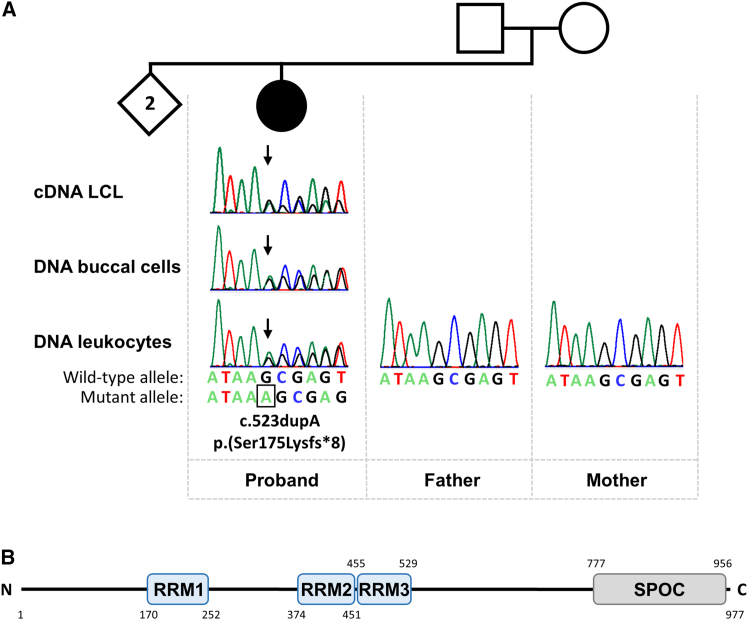
*De novo RBM15* frameshift variant in the proband with mirror movements, *RBM15* transcript analysis, and RBM15 domain structure (A) The top image displays the pedigree of the proband’s family. The second top image shows an electropherogram of wild-type and variant *RBM15* transcripts in cDNA derived from the proband’s lymphoblastoid cell line (LCL). The middle and bottom images show partial sequence electropherograms demonstrating the heterozygous *RBM15* frameshift variant (c.523dupA [GenBank: NM_022768.5] [p.Ser175Lysfs∗8]) in genomic DNA isolated from the proband’s leukocytes and buccal cells, respectively. The *RBM15* variant was absent in leukocyte-derived DNA from her healthy parents (bottom). The sequence of the wild-type and mutant alleles is given below the proband’s electropherograms. The duplicated adenine is highlighted by a rectangle, and an arrow points to the start of the frameshift in the electropherograms. (B) Domain structure of RBM15 according to UniProt database (UniProt: Q96T37). The RNA recognition motifs (RRM1–3) are represented by blue boxes and the Spen paralog and ortholog C-terminal (SPOC) domain by a gray box. Amino acid numbering is given according to the reference sequence GenBank: NP_073605.4.

We describe a 27-year-old female with mild mirror movements affecting only the hands (Table 1; see the supplemental note and Figure S1 for the full clinical description). To identify the genetic cause in the proband, we performed trio exome sequencing in the proband and healthy parents. We did not detect any rare, likely pathogenic variant in any of the known causative genes for CMMs, such as *DCC*, *NTN1*, *RAD51*, or *ARHGEF7*. However, we identified a *de novo* heterozygous 1-bp duplication, c.523dup (p.Ser175Lysfs∗8), in *RBM15* (MANE Select transcript: NM_022768.5) in the proband that was confirmed by Sanger sequencing in DNA isolated from the proband’s leukocytes and buccal cells (Figure 1A). The variant was absent in the proband’s parents (Figure 1A). The c.523dup variant is predicted to cause a frameshift and the introduction of a premature stop codon, p.Ser175Lysfs∗8, and thus is likely a heterozygous LoF variant. To assess whether the *RBM15* variant leads to nonsense-mediated mRNA decay (NMD) of variant transcripts, we performed RT-PCR followed by Sanger sequencing using RNA (cDNA) isolated from a lymphoblastoid cell line of the proband. Both wild-type and *RBM15* transcripts containing the 1-bp duplication were detected, with sequence traces of the wild-type mRNAs approximately twice as abundant as those of the variant-containing mRNAs (Figure 1A). These findings suggest only partial NMD of the mutant *RBM15* mRNA in the proband’s cells, potentially due to the variant’s location within the large exon 1 (2,892 bp) of the gene.

**Table 1:** Phenotypic findings mapped to HPO terms in the proband with the heterozygous *de novo RBM15* variant

	Proband
Genotype	c.523dup (GenBank: NM_022768.5) (p.Ser175Lysfs∗8)
Autosomal dominant inheritance (HP: 0000006)	+
Sex	female
Age	27 years
Pregnancy and birth	unremarkable
Childhood onset (HP: 0011463)	+
Bimanual synkinesia (HP: 0001335)	+
Abnormal corticospinal tract morphology (HP: 0002492)	+

+, present.

The frameshift variant is absent in the gnomAD database (v.4.1.0)^17^ and in the Regeneron Genetics Center Million Exome data.^18^ *RBM15* is intolerant to LoF variants, as the LOEUF (LoF observed/expected upper bound fraction) is 0.208 and the pLI (probability of being loss-of-function intolerant) score is 1 (gnomAD v.4.1.0). Thus, *RBM15* is a highly likely haploinsufficient gene.^17^ Similarly, the four autosomal-dominant disease genes for CMMs are also intolerant to LoF variants, as they have a LOEUF between 0.4 and 0.5 and, except for *ARHGEF7*, a pLI score of 1.^17^ Together, based on the absence of the *RBM15* variant p.Ser175Lysfs∗8 in population databases and *de novo* occurrence of the variant in the proband, the results show that the variant may underlie CMMs in the female. We received five matches on heterozygous *RBM15* variants from GeneMatcher^19^; however, the phenotypes do not fit our proband, and/or another genetic cause was identified in the affected individuals.

**Figure 2:**
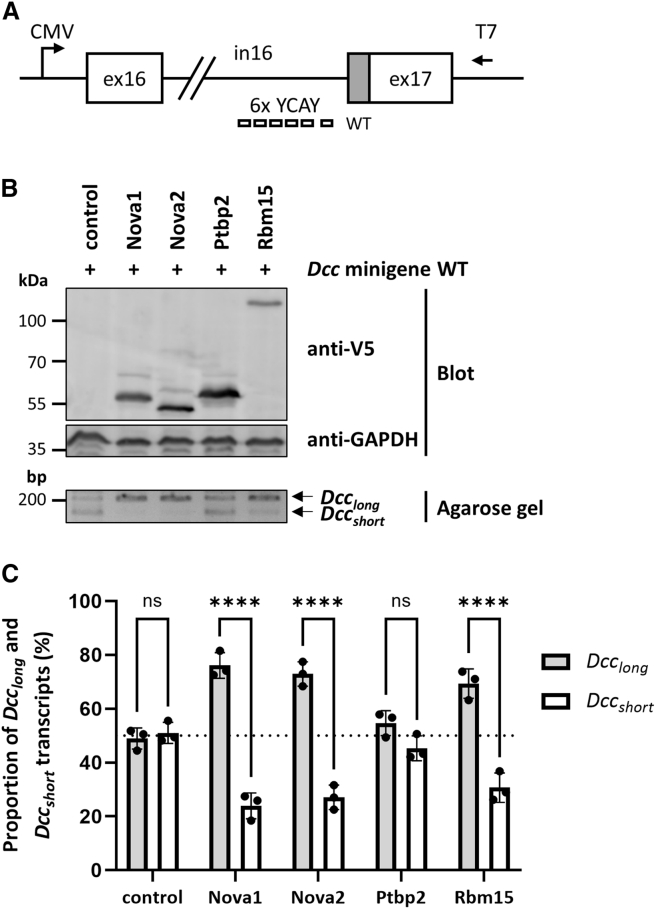
Rbm15 regulates alternative splicing of *Dcc* exon 17 (A) Schematics of the *Dcc* minigene construct with the genomic DNA of mouse *Dcc* exons 16 and 17 and the 5.2-kb intron 16. The CMV promoter and the position of the T7 primer binding site are indicated. The YCAY cluster (Y = C/U) composed of six YCAY repeats (rectangles) in intron 16 is shown. The alternatively spliced sequence of exon 17 (60 bp) is highlighted in gray. The figure is adapted from Figure 9 in Leggere et al.^14^ (B) HEK293T cells were transfected with the *Dcc* minigene construct together with pCAGGS plasmids expressing V5-tagged mouse Nova1, Nova2, Ptbp2, or Rbm15 or with an empty pCAGGS vector as control. After 48 h, lysates and total RNA were prepared. Expression of V5-tagged proteins was monitored with an anti-V5 antibody. An anti-GAPDH antibody was used to demonstrate equal loading. Total RNA was reverse transcribed from the T7 promoter, and semi-quantitative PCR was performed to simultaneously amplify *Dcc*_*long*_ and *Dcc*_*short*_ transcripts. A representative blot and a representative image of a 2% agarose gel are shown in the top and bottom images, respectively. (C) Band intensities of the two PCR products were quantified by densitometric analysis. The relative abundance of *Dcc*_*long*_ and *Dcc*_*short*_ transcripts is shown, with the sum of both signal intensities set to 100%. The graph bars represent the mean ± SD of three independent experiments (*n* = 3). Individual data points and a grid line at 50% are shown. A two-way ANOVA followed by Šidák post hoc test was used for statistical analysis. ex, exon; in, intron; ns, not significant; WT, wild type; ∗∗∗∗*p* ≤ 0.0001.

RBM15 is an RNA-binding protein and consists of three N-terminal RNA recognition motifs (RRMs) and a Spen paralog and ortholog C-terminal (SPOC) domain (Figure 1B). Serine 175, affected by the variant, is located in the RRM1 domain (Figure 1B). RBM15 is implicated in multiple biological processes, such as transcription, RNA modification, RNA export, X chromosome inactivation, and alternative splicing.^20^ As part of a protein complex that regulates alternative splicing, RBM15 is localized in nuclear speckles, which are nuclear structures enriched in pre-mRNA splicing factors.^21^ Alternative splicing of RBM15 target genes is mediated by the binding of RBM15 to specific intronic regions and recruitment of the splicing factor SF3B1 to pre-mRNA molecules.^22^ The function of RBM15 in alternative splicing led us to hypothesize that this RNA-binding protein may regulate alternative splicing of *DCC* pre-mRNAs. We tested this hypothesis by carrying out an *in vitro* splicing assay. We used a previously published minigene construct that contained the genomic DNA between exons 16 and 17 of mouse *Dcc* (Figure 2A). The first 60 bp of *Dcc* exon 17 are alternatively spliced, and Nova1 and Nova2 recognize six clusters of the DNA sequence YCAY (Y = C/U) in intron 16 to promote the expression of *Dcc*_*long*_ transcripts, while the amount of *Dcc*_*short*_ mRNAs is reduced.^14^ We co-transfected HEK293T cells with the *Dcc* minigene construct and a construct expressing V5-tagged Nova1, Nova2, Rbm15, Ptbp2 (a splicing factor that does not regulate *Dcc* alternative splicing), or the empty vector (pCAGGS-V5). We confirmed expression of Nova1, Nova2, Rbm15, and Ptbp2 proteins by immunoblotting (Figure 2B). Co-expression of Nova1-V5 or Nova2-V5 with the *Dcc* minigene construct, followed by RT-PCR to amplify a *Dcc* exon 16–17 product, revealed a strong band corresponding to *Dcc*_*long*_ and a weak *Dcc*_*short*_ band (Figure 2B). Quantification of band intensities revealed proportions of *Dcc*_*long*_ and *Dcc*_*short*_ of ∼76:24 and ∼73:27 for Nova1 and Nova2, respectively (Figure 2C). In contrast, transfection of HEK293T cells with the empty V5 vector or the vector expressing Ptbp2-V5 resulted in similar levels of both *Dcc* transcript variants (Figure 2B), with proportions of *Dcc*_*long*_ and *Dcc*_*short*_ of ∼49:51 and ∼55:45 for the empty vector and Ptbp2, respectively (Figure 2C). When Rbm15 was expressed, we observed a similar effect as for Nova1 and Nova2: the band corresponding to *Dcc*_*long*_ was strong, while the one for *Dcc*_*short*_ was weak (Figure 2B). Quantitative analysis revealed proportions of *Dcc*_*long*_ and *Dcc*_*short*_ of ∼69:31 (Figure 2C). Together, the data suggest that Rbm15, similar to Nova1 and Nova2, promotes inclusion of the entire exon 17 in the *Dcc* mRNA to express *Dcc*_*long*_.

## Discussion

We report here on a female with mirror movements of the hands and a heterozygous *de novo* frameshift variant, c.523dup (p.Ser175Lysfs∗8), in *RBM15* that likely is a LoF allele. Transcranial magnetic stimulation results support the existence of abnormal ipsilateral corticospinal projections in addition to normally crossed corticospinal projections in the affected individual. Similarly, an abnormal uncrossed corticospinal tract has been observed in individuals with CMMs and a heterozygous *DCC*, *RAD51*, or *NTNT1* variant.^1^^,^^6^^,^^23^ These data suggest that heterozygous *RBM15* LoF variants may be associated with CMMs and that RBM15 may have a function in axon guidance in the corticospinal tract.

In the spinal cord, commissural neurons extend their axons toward and across the ventral midline by integrating attractive and repulsive signals. Among the attractive cues, Netrin-1 and its receptor Dcc play key roles. Netrin-1 is an extracellular protein that binds to Dcc to guide many commissural axons within the central nervous system.^2^ Depletion of Netrin-1 from the floor plate and the ventricular zone results in a failure of corticospinal tract midline crossing and leads to mirror movements in mice.^24^^,^^25^ In mice mutants with a conditional deletion of *Dcc* in the corticospinal tract, however, the tract’s anatomy remains normal, suggesting that Dcc may function in a non-cell-autonomous manner.^23^

Alternative splicing of *Dcc* exon 17 is mediated by Nova1 and Nova2, which promote the production of a Dcc_long_ isoform and reduce the production of Dcc_short_ to control commissural axon attraction.^14^ A double *Nova1/2* knockout in the mouse spinal cord causes a failure in neuronal migration, axon outgrowth, and axon guidance of commissural interneurons, a phenotype that is similar to *Dcc* knockout. Only Dcc_long_, not Dcc_short_, rescues the axon projection defect in the Nova1/2 double knockout.^14^ A recent study has demonstrated that the two Dcc isoforms have distinct functions, with Dcc_short_ being unable to activate Netrin-1-induced signaling, and both are required for commissural axon midline crossing. While Dcc_long_ facilitates axon growth and midline entry, Dcc_short_ reduces axon growth and allows commissural axons to exit the midline. Thus, spatially and temporally regulated expression of Dcc_long_ and Dcc_short_ during commissural neurogenesis is important for fine-tuning Netrin-1 signaling around the midline.^26^ To date, RBM15-regulated alternative splicing has only been shown to be important for megakaryocyte differentiation.^22^ Different conditional deletions of *Rbm15* in mice have demonstrated Rbm15’s function in the development of the hematopoietic system, heart, and spleen,^27^^,^^28^ while homozygous *Rbm15* germline deletion causes embryonic lethality.^28^ Importantly, Rbm15 also has a role in the nervous system, such as in normal development of the mouse cerebral cortex^29^ and in controlling axon outgrowth and branching and other processes to establish developmental plasticity of neurons in *Drosophila*.^30^ Human *RBM15* is highly expressed in many brain regions during nervous system development, with expression levels decreasing postnatally, beginning as early as 4 months of age. However, relatively high *RBM15* expression persists in the cerebellar cortex until 30–37 years of age (Figure S2). Based on these data, it will be important to study Rbm15’s function in the corticospinal tract and whether Rbm15 is required for migration, axon outgrowth, and/or guidance of commissural neurons. For example, *Rbm15* knockout or knockdown studies in the spinal cord using whole mouse embryo culture are required to show a potential role of Rbm15 in midline crossing of axons and in the regulation of *Dcc* alternative splicing. Moreover, the identification of additional individuals with CMMs and heterozygous *RBM15* variants is important to provide further evidence for the provisional Mendelian gene discovery reported here.

## Data and code availability

The published article includes all data generated or analyzed during this study. Consent restrictions preclude sharing of full data sets, and the consents do not cover the deposition of the exome sequencing data in a public database. *RBM15* variant and phenotypic information were submitted to the LOVD database (https://databases.lovd.nl/shared/genes/RBM15), with the LOVD Variant ID #0001030018.

## Acknowledgments

We are grateful to the proband and parents who agreed to participate in this project. We thank Lara Adrian and Henrike Wilshusen for skillful technical assistance. This work was supported by the 10.13039/501100001659Deutsche Forschungsgemeinschaft (KU 1240/17-1 to K.K.).

## Declaration of interests

The authors declare no competing interests.

## Web resources

GenBank, https://www.ncbi.nlm.nih.gov/genbank/
GeneMatcher, https://genematcher.org/
gnomAD, https://gnomad.broadinstitute.org
Human Phenotype Ontology (HPO), https://hpo.jax.org/
Online Mendelian Inheritance in Man (MIM), http://www.omim.org/
Regeneron Genetics Center Million Exome Variant Browser, https://rgc-research.regeneron.com/me/home
UniProt, https://www.uniprot.org/

## Supplemental information

Document S1. Figures S1 and S2, Table S1, supplemental note, and supplemental material and methods

Document S2. Article plus supplemental information
