# Supplementary material for: A de novo frameshift variant in the candidate RBM15 in a proband with congenital mirror movements
Source: HGG Adv. 2025 Oct 7;7(1):100528. doi: 10.1016/j.xhgg.2025.100528 (PMC12554026; doi:10.1016/j.xhgg.2025.100528)
Supplement: Document S1. Figures S1 and S2, Table S1, supplemental note, and supplemental material and methods [file mmc1.pdf]

HGGA, Volume 7

Supplemental information

A *de novo* frameshift variant in the candidate *RBM15* in a proband with congenital mirror movements

Frederike L. Harms, Fanny Kortüm, Malik Alawi, Martin Staudt, and Kerstin Kutsche

Supplemental Information

Supplemental case report

We describe a 27-year-old female with mirror movements. The proband is the third child of healthy non-consanguineous parents. She has two elder siblings, one is healthy, while the other one was diagnosed with multiple sclerosis. The proband's pregnancy and birth were uneventful. Motor development was slightly delayed (independent walking at >18 months of age), other milestones were reached at appropriate ages. At the age of 3-4 years, the parents noticed their daughter's pronounced mirror movements while drawing.

At the age of 7 years, she was referred to M.S. (Department of Pediatric Neurology and Developmental Medicine, University Children's Hospital Tübingen, Germany) for the evaluation of mirror movements. Her parents had noticed that during intentional movements of one hand, the other hand performed involuntary movements "mirroring" the intended movements in the task hand. These mirror movements could be suppressed, at least to some degree, by voluntarily increasing the muscle tone in the non-task hand, but complete suppression was not possible. Apart from mirror movements, standard neurological examination was normal, and cranial MRI was also normal. Transcranial magnetic stimulation (TMS; MagStim 200; focal figure-eight-coil; bilateral registration of motor evoked potentials from M. interosseus dorsalis I via surface EMG electrodes), however, demonstrated abnormalities in the organization of the corticospinal tract, with prominent fast-conducting ipsilateral projections: TMS of the right hemisphere elicited motor evoked potentials (MEPs) in both hands (80% stimulator output; no pre-contraction), with latencies of 17.1 ms (left hand) and 17.7 ms (right hand). Similarly, TMS of the left hemisphere (100% stimulator output) also elicited MEPs in both hands (no pre-contraction), with latencies of 17.4 ms (right hand) and 18.3 ms (left hand).

At the age of 20 years, she was again evaluated by M.S. (Center for Pediatric Neurology and Neurorehabilitation, Schön Clinic Vogtareuth, Germany). The proband reported only minimal problems during activities of daily living: after long periods of writing or drawing by hand (with her right hand), the muscle tone in her (non-writing) left hand increased slowly and sometimes caused minor pain. The

proband used to play the trumpet for several years, but had stopped playing. She tried playing the piano, but it was not possible. She was a good volleyball-player for a period of 6-7 years. In school, she described herself as talented in languages, but rather weak in mathematics, where she had required additional support to obtain the secondary school certificate. Neurological examination revealed marked mirror movements during some activities (e.g. untying shoelaces), which were less marked but still present in the artificial situation of a neurological examination, e.g. during flexing / extending fingers and pro- / supination. No mirror movements were observed in the more proximal joints of the upper extremity and in the lower extremity. When nervous, the proband showed minimal intention tremor (left > right), brisk deep tendon reflexes, but negative pyramidal tract signs. TMS of the right hemisphere elicited MEPs not only in the contralateral left hand (resting motor threshold (RMT): 33%, latency at 110% RMT: 21.0 ms), but also in the right hand (RMT 50%, latency at 110% RMT: 20.6 ms). TMS of the left hemisphere elicited MEPs not only in the contralateral right hand (resting motor threshold (RMT): 36%, latency at 110% RMT: 21.2 ms), but also in the left hand (RMT 42%, latency at 110% RMT: 22.2 ms). Thus, TMS examinations at 7 and 20 years of age demonstrated that both hemispheres of the proband had normally crossed corticospinal projections and abnormal ipsilateral corticospinal projections (requiring somewhat higher stimulation intensities than for the crossed projections – this could be demonstrated in the second TMS study, where a more cooperative proband allowed a more detailed examination).

At the age of 26 years, the proband experienced sensory disturbances affecting the left arm and the left side of the face, which prompted her hospital admission. Examinations excluded intracranial and spinal causes, as well as inflammatory central nervous system diseases, as the underlying source of her symptoms. Brain MRI revealed unremarkable infratentorial and supratentorial findings (**Supplemental Figure 1**).

In a telephone interview, the 27-year-old proband reported no current limitations in her daily living (except for the inability to play the piano). She had continued her school career and obtained the advanced technical college certificate, went to college and graduated as a specialist for media and

information technology. In her job she works in an office and uses her own “6-finger typing system”.

She had tried to learn the 10-finger typing system, but has not been successful.

Supplemental figures and legends

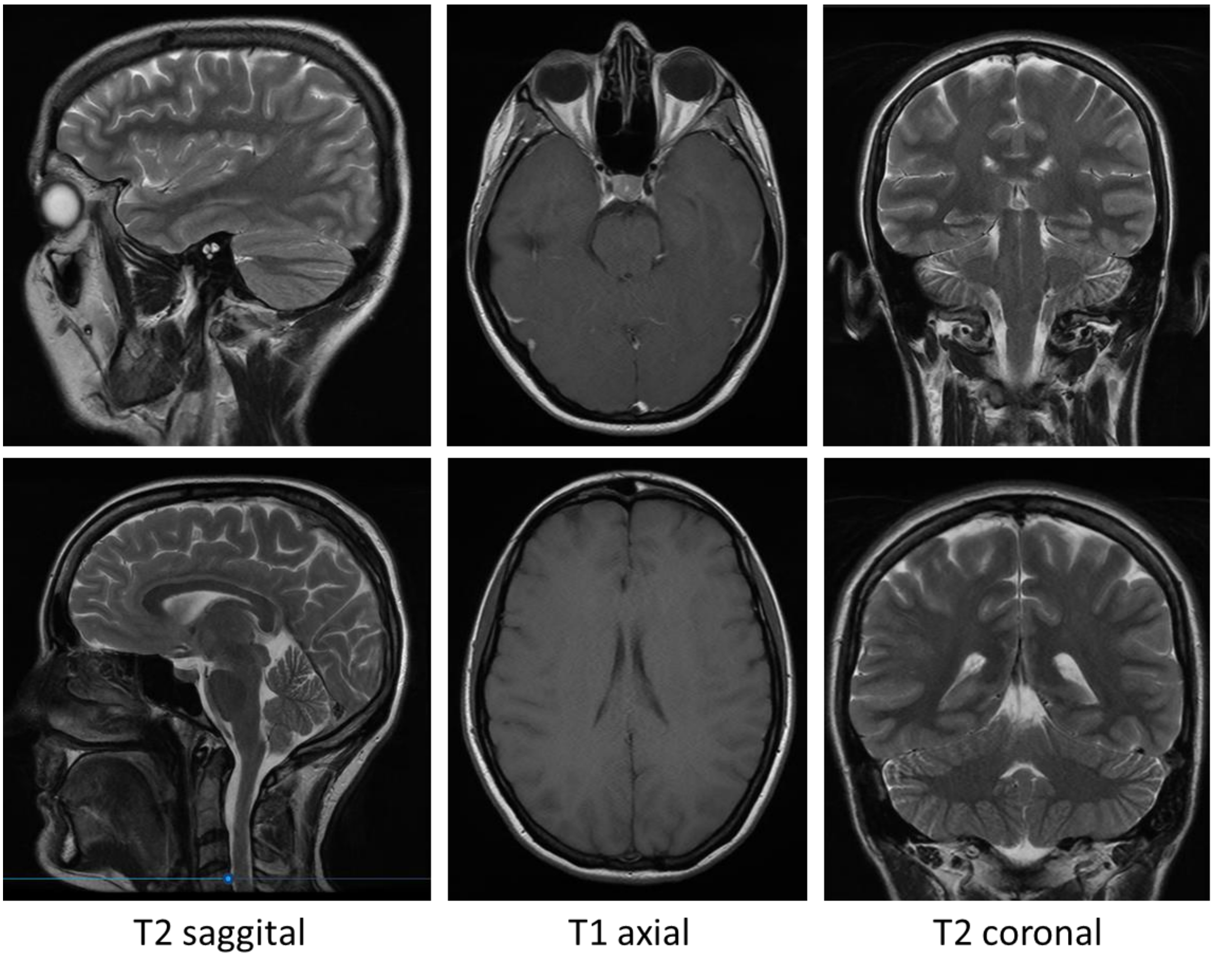

Supplemental Figure 1. Selected brain MRI scans of the proband at the age of 26 years

Sagittal (left) and coronal (right) T2-weighted images, as well as axial (middle) T1-weighted images, show unremarkable infratentorial and supratentorial findings.

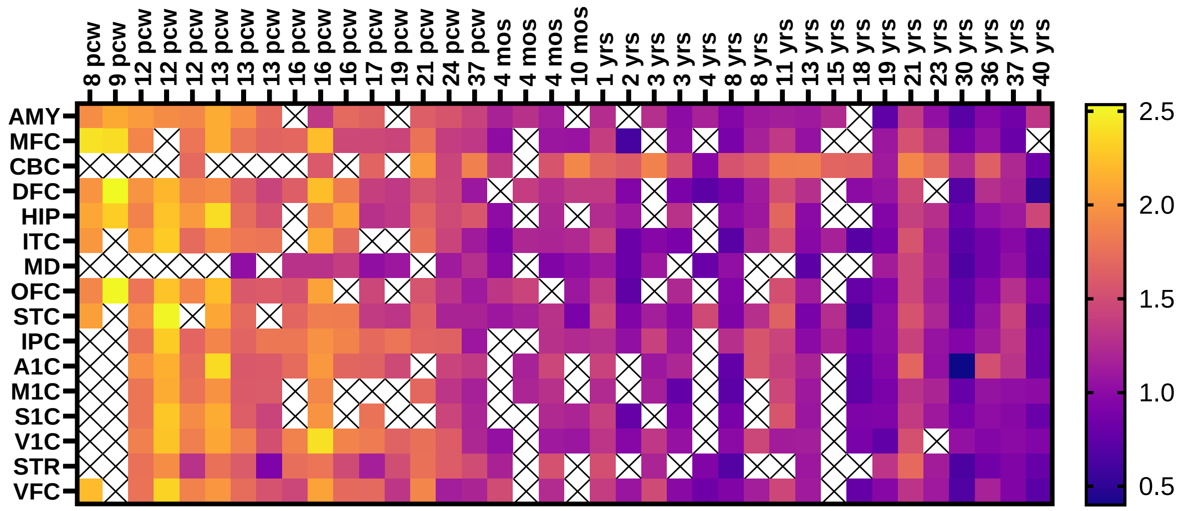

Supplemental Figure 2. Spatio-temporal expression of *RBM15* mRNA in the human brain

The heatmap illustrates relative *RBM15* mRNA expression across human brain samples from donors aged 8 to 37 post-conception weeks (pcw) and from 4 months (mos) to 40 years (yrs). Expression was analyzed in the following brain regions: amygdaloid complex (AMY), anterior (rostral) cingulate (medial prefrontal) cortex (MFC), cerebellar cortex (CBC), dorsolateral prefrontal cortex (DFC), hippocampus (HIP), inferolateral temporal cortex (ITC), mediodorsal thalamic nucleus (MD), orbital frontal cortex (OFC), posterior (caudal) superior temporal cortex (STC), posteroventral (inferior) parietal cortex (IPC), primary auditory cortex (A1C), primary motor cortex (M1C), primary somatosensory cortex (S1C), primary visual cortex (V1C), striatum (STR), and ventrolateral prefrontal cortex (VFC). Dark violet indicates low expression and yellow high expression (log_2 RPKM (reads per kilobase of transcript); scale shown on the right). Data were obtained from the *BrainSpan Atlas of the Developing Human Brain* (© 2016 Allen Institute for Brain Science. Aging, Dementia and TBI study; available at: https://www.brainspan.org/).[4]

Supplemental Table

Supplemental Table 1. Sequence of oligonucleotides used in this work

***RBM15* primer sequences for variant validation**			
**Template**	**Exon**	**Direction**	**Sequence (5' → 3')**
DNA	1	forward	GTG GGG GCA GCT CCA GTA
		reverse	AGA GGC CGG TCA TAG AGC AC
***RBM15* primer sequences for transcript analysis (RT-PCR)**			
**Template**	**Exon**	**Direction**	**Sequence (5' → 3')**
cDNA	1	forward	GCT CCA GTA GCC GCT TGC
	2	reverse	AAC TCC TAT CTG AAA ACC AAA CC
**Primer sequences for cloning the mouse *Rbm15* coding region into pCAGGS-MCS-V5**			
**Template**		**Direction**	**Sequence (5' → 3')**
cDNA from mouse brain		forward	TTG GCA AAG AAT TCG GTA CCA TGA GGT CTG CGG GGC GG
		reverse	GTG CTC GAG GCG GCC TCC GCT GTT CAC CAG TTT TGC
**Primer sequences for *Dcc* minigene assay**			
**Task**		**Direction**	**Sequence (5' → 3')**
Reverse transcription from T7 promotor		reverse	CTA TAG TGA GTC GTA TTA
Amplification of Dcc_{long} and Dcc_{short} transcripts		forward	TCT CAT TAT GTA ATC TCC TTA AAA GC
		reverse	TCA CAG CCT CAT GGG TAA GAG

Supplemental methods

Exome sequencing

DNA was isolated by standard procedures. Trio exome sequencing (ES) was performed on leukocyte-derived DNA from the proband and her parents as described previously.[1] Briefly, enrichment was carried out using the Nextera Enrichment kit (62 Mb) (Illumina). Captured libraries were then loaded and sequenced on the HiSeq2000 platform (Illumina, San Diego, CA). Trimmomatic was employed to remove adapters, low quality (phred quality score < 5) bases from the 3' ends of sequence reads.[2] Reads shorter than 36 bp were subsequently removed. Further processing was performed following the Genome Analysis Toolkit's (GATK) best practice recommendations. Briefly, trimmed reads were aligned to the human reference genome (UCSC GRCh37/hg19) using the Burrows-Wheeler Aligner (BWA mem v0.7.12). Duplicate reads were marked with Picard tools (v1.141). GATK (v3.4) was employed for indel realignment, base quality score recalibration, calling variants using the HaplotypeCaller, joint genotyping, and variant quality score recalibration. AnnoVar (v2015-03-22) was used to functionally annotate and filter alterations against public databases (dbSNP138, 1000 Genomes Project, and ExAC Browser). Only exonic and intronic variants that were *de novo* (absent from public databases) or rare (with a minor allele frequency [MAF] ≤0.1% and no homo- and hemizygotes in public databases) were retained. Variants with poor depth of sequencing coverage (total read depth < 10) and in low quality regions (checked in IGV) were discarded.

Variant validation

RBM15 variant validation was performed by Sanger-sequencing using leukocyte-derived DNA from the proband and parents and DNA isolated from buccal cells of the proband. Primers designed to amplify the selected region of *RBM15* exon 1 (NM_022768.5) are described in **Supplemental Table 1**. Amplicons were directly sequenced using the ABI BigDye Terminator Sequencing Kit (Applied Biosystems) and an automated capillary sequencer (ABI 3500, Applied Biosystems). Sequence electropherograms were analyzed using Chromas v2.6.6 (Technelysium Pty Ltd).

Plasmid information and cloning procedures

pCAGGS-Nova1-V5, pCAGGS-Nova2-V5, pCAGGS-Ptbp2-V5, pCAGGS-V5 empty vector, and wild-type and mutant *Dcc* minigene constructs (1xYCAY mut, 4xYCAY mut, 5xYCAY mut, and 6xYCAY mut) in pDEST26 backbones were kindly provided by Harald J. Junge (Department of Ophthalmology and Visual Neurosciences, University of Minnesota, Minneapolis, MN 55455, USA) and Zhe Chen (Department of Neuroscience, University of Minnesota, Minneapolis, MN 55455, USA).[3]

To generate a construct for expression of C-terminally V5-tagged Rbm15 (pCAGGS-Rbm15-V5), we amplified the coding region of mouse *Rbm15* (NM_001045807.2) using primers and cDNA of mouse brain as a template. The purified PCR product was then cloned between the *Kpn*I and *Not*I restriction sites of pCAGGS-V5 using the InFusion HD Cloning Kit (Takara). All constructs were regularly sequenced for integrity and primer sequences for InFusion cloning are described in **Supplemental Table 1**.

Cell culture conditions

HEK293T were cultured in Dulbecco's modified Eagle medium (DMEM; Thermo Fisher Scientific) supplemented with 10% fetal bovine serum (FBS; GE Healthcare) and penicillin-streptomycin (100 U/mL and 100 mg/mL, respectively; Thermo Fisher Scientific). The proband-derived lymphoblastoid cell line was maintained in RPMI 1640 medium (Gibco) supplemented with 20% FBS and penicillin-streptomycin.

***RBM15* transcript analysis**

Total RNA was extracted from proband-derived lymphoblastoid cells using the RNeasy Mini Kit (Qiagen). 1 µg total RNA was reverse transcribed using oligo(dT) primer, and semi-quantitative RT-PCR was performed to amplify *RBM15* transcripts (primer sequences are listed in **Supplemental Table 1**). The resulting RT-PCR products were directly Sanger sequenced using the forward primer.

***Dcc* minigene assay**

The *Dcc* minigene assay was adapted from Leggere *et al.* (2016).[3] Briefly, HEK293T cells were transiently transfected with wild-type or mutant *Dcc* minigene construct together with pCAGGS-Nova1-V5, pCAGGS-Nova2-V5, pCAGGS-Ptbp2-V5, pCAGGS-Rbm15-V5, or pCAGGS-V5 empty vector using TurboFect transfection reagent (Thermo Fisher Scientific) following the manufacturer's protocol. Transfection medium was changed 4-6 hours after transfection and cells were cultured in 10% DMEM for 48 h. Subsequently, total RNA was extracted using RNeasy Mini Kit (Qiagen) and whole-cell lysates were collected in ice-cold RIPA buffer [50 mM Tris-HCl, pH 8.0; 150 mM NaCl; 1% NP-40; 0.5% DOC (sodium deoxycholate); 0.1% SDS (sodium dodecyl sulfate)] supplemented with Mini Protease Inhibitor (Roche).

The concentration and purity of the RNA samples were assessed using the Epoch™ Microplate Spectrophotometer (BioTek). 1 µg total RNA was reverse transcribed from the T7 promoter and semi-quantitative PCR was performed to amplify Dcc_{long} and Dcc_{short} transcripts (for primer sequences see **Supplemental Table 1**). For analysis, PCR products were separated on a 2% agarose gel and band intensities were quantified by densitometric analysis using the ImageJ software.

Expression of V5-tagged proteins in whole-cell lysates was confirmed with immunoblotting using mouse monoclonal anti-V5 (Invitrogen; #R960-25; clone SV5-Pk1; 1:5,000 dilution) as primary and horseradish peroxidase-conjugated sheep anti-mouse (GE Healthcare; NA931V; 1:10,000 dilution) as secondary antibody. For control of equal loading, whole-cell lysates were analyzed using a mouse monoclonal anti-GAPDH antibody (Abcam; #ab8245; 1:10,000 dilution). Immunoblots were digitally imaged using a ChemiDoc system (Bio-Rad), with exposure time optimized to avoid saturation.

Data analysis and statistics

Quantitative data are presented by GraphPad Prism 8 software (Instat, GraphPad Software) as the mean ± standard deviation (SD). For quantification, two-way ANOVA followed by a Šidák *post hoc* test for multiple comparisons was performed. A *p*-value ≤ 0.05 was considered statistically significant.

Supplemental references

1. Kortum, F., Caputo, V., Bauer, C.K., Stella, L., Ciolfi, A., Alawi, M., Bocchinfuso, G., Flex, E., Paolacci, S., Dentici, M.L., et al. (2015). Mutations in KCNH1 and ATP6V1B2 cause Zimmermann-Laband syndrome. Nat Genet *47*, 661-667. 10.1038/ng.3282.
2. Bolger, A.M., Lohse, M., and Usadel, B. (2014). Trimmomatic: a flexible trimmer for Illumina sequence data. Bioinformatics *30*, 2114-2120. 10.1093/bioinformatics/btu170.
3. Leggere, J.C., Saito, Y., Darnell, R.B., Tessier-Lavigne, M., Junge, H.J., and Chen, Z. (2016). NOVA regulates Dcc alternative splicing during neuronal migration and axon guidance in the spinal cord. Elife *5*, e14264. 10.7554/eLife.14264.
4. Miller, J.A., Guillozet-Bongaarts, A., Gibbons, L.E., Postupna, N., Renz, A., Beller, A.E., Sunkin, S.M., Ng, L., Rose, S.E., Smith, K.A., et al. (2017). Neuropathological and transcriptomic characteristics of the aged brain. Elife *6,* e31126. 10.7554/eLife.31126.
