# Supplementary material for: A de novo frameshift variant in the candidate RBM15 in a proband with congenital mirror movements
Source: HGG Adv. 2025 Oct 7;7(1):100528. doi: 10.1016/j.xhgg.2025.100528 (PMC12554026; doi:10.1016/j.xhgg.2025.100528)
Supplement: Document S2. Article plus supplemental information [file mmc2.pdf]

HGG Advances

BRIEF COMMUNICATION

A *de novo* frameshift variant in the candidate *RBM15* in a proband with congenital mirror movements

Frederike L. Harms,[1] Fanny Kortüm,[1] Malik Alawi,[2] Martin Staudt,[3] and Kerstin Kutsche[1,4,5,*]

Summary

Congenital mirror movements (CMMs) are involuntary movements of one side of the body that mirror intentional movements of the opposite side. *DCC*, *NTN1*, *RAD51*, *ARHGEF7*, and *DNAL4* have been associated with CMMs. Two-thirds of CMM-affected individuals remain without a genetic diagnosis, indicating that variants in additional genes need to be discovered. We report on a 27-year-old female with CMMs of the hands. Trio exome sequencing in the proband and healthy parents did not reveal a likely pathogenic variant in one of the CMM-associated genes but rather a *de novo* heterozygous frameshift variant c.523dup (p.Ser175Lysfs*8) in the candidate *RBM15*. The variant results in only partial nonsense-mediated mRNA decay of *RBM15* transcripts in the proband's lymphoblastoid cells. *RBM15* encodes an RNA-binding protein involved in alternative splicing as well as other processes. *Dcc* alternative splicing generates Dcc_{long} and Dcc_{short} isoforms, which are important for commissural axon midline crossing. We tested whether Rbm15 regulates *Dcc* alternative splicing by using an *in vitro* minigene assay. Ectopic expression of Rbm15, similar to the splicing factors Nova1 and Nova2, promotes the production of Dcc_{long} transcripts. The possible link between Rbm15 and Dcc supports a role for Rbm15 in CMMs.

Introduction

Congenital mirror movements (CMMs) are involuntary movements of one side of the body that mirror voluntary movements of the opposite side. The onset of CMMs is in infancy or early childhood; they persist throughout life and vary in severity. The upper limbs, especially the fingers, are predominantly affected, resulting in difficulty with activities of daily living. CMMs are associated with abnormal ipsilateral corticospinal tract and bilateral activation of the primary motor cortices during movement preparation and execution.[1–3] Thus, abnormal ipsilateral corticospinal tract and bilateral activation of the primary motor cortices likely underlie mirror movements.[1,2]

CMMs can occur in simplex cases or are inherited in an autosomal-dominant fashion with incomplete penetrance in most of the families. It is a genetically heterogeneous disorder and is caused by heterozygous pathogenic variants in *DCC* (MIM: 120470),[4] *RAD51* (MIM: 179617),[5] *NTN1* (MIM: 601614),[6] and *ARHGEF7* (MIM: 605477),[7] while a homozygous splice-site variant in *DNAL4* has been reported in a single Pakistani family.[8] Genetic analysis in large cohorts of individuals with CMMs identified *DCC* as the most commonly mutated gene, while pathogenic variants in *RAD51*, *NTN1*, and *ARHGEF7* were rarely found in affected individuals.[9–11] Overall, an underlying genetic cause can be found in about one-third of the affected individuals, with a yield of 70% in those with a positive family history. Thus, the high proportion of individuals with CMMs that are genetically unsolved suggests the existence of additional genes associated with CMMs that need to be discovered.[9] The lack of reports on novel CMM-associated genes for several years and the identification of pathogenic variants in *DNAL4* and *ARHGEF7* in single families suggest a high degree of genetic heterogeneity, with affected simplex individuals and families possibly carrying a pathogenic variant in their private gene.

DCC, NTN1 (Netrin-1), and ARHGEF7 are required for Netrin-1-stimulated commissural axon outgrowth and guidance.[2,7] The secreted protein Netrin-1 binds to the DCC receptor,[12,13] and ARHGEF7 is important for Netrin-1-induced increase of DCC at the cell surface of commissural neuron growth cones.[7] The DCC receptor is a transmembrane protein of the immunoglobulin superfamily of cell adhesion molecules.[12] Alternative splicing of the first 60 bp of *Dcc* exon 17 generates two isoforms that differ in 20 amino acid residues in the linker region between the fourth and fifth fibronectin repeats, referred to as Dcc_{long} and Dcc_{short}.[14,15] The RNA-binding proteins Nova1 and Nova2 regulate alternative splicing of *Dcc*. *Dcc* alternative splicing is perturbed in a *Nova1*/*Nova2* double-knockout mouse that shows commissural axon guidance defects, demonstrating that alternative splicing of *Dcc* exon 17 is important for spinal commissural neuron development.[14,16]

Here, we report on a female with CMMs and no pathogenic variant in any of the known genes associated with CMMs, such as *DCC*, *RAD51*, *NTN1*, and *ARHGEF7*. Trio exome sequencing in the proband and parents identified a *de novo* frameshift variant in the candidate gene

[1]Institute of Human Genetics, University Medical Center Hamburg-Eppendorf, 20246 Hamburg, Germany; [2]Bioinformatics Core, University Medical Center Hamburg-Eppendorf, 20246 Hamburg, Germany; [3]Center for Pediatric Palliative Care, Dr. von Hauner Children's Hospital, Ludwig-Maximilian-University, Campus Grosshadern, 81377 Munich, Germany; [4]German Center for Child and Adolescent Health (DZKJ), Partner Site Hamburg, 20246 Hamburg, Germany
[5]Lead contact
*Correspondence: kkutsche@uke.de
https://doi.org/10.1016/j.xhgg.2025.100528.
© 2025 The Authors. Published by Elsevier Inc. on behalf of American Society of Human Genetics.
This is an open access article under the CC BY license (http://creativecommons.org/licenses/by/4.0/).

Table 1. Phenotypic findings mapped to HPO terms in the proband with the heterozygous *de novo RBM15* variant

	Proband
Genotype	c.523dup (GenBank: NM_022768.5) (p.Ser175Lysfs*8)
Autosomal dominant inheritance (HP: 0000006)	+
Sex	female
Age	27 years
Pregnancy and birth	unremarkable
Childhood onset (HP: 0011463)	+
Bimanual synkinesia (HP: 0001335)	+
Abnormal corticospinal tract morphology (HP: 0002492)	+

+, present.

RBM15 (MIM: 606077) that likely is a loss-of-function (LoF) allele. Our *in vitro* studies revealed a role for the RNA-binding protein Rbm15 in alternative splicing of *Dcc* by promoting the expression of Dcc_{long} transcripts, similar to Nova1 and Nova2 splicing factors. Our data suggest that Rbm15 may be involved in regulating the production of the functionally distinct Dcc_{long} and Dcc_{short} isoforms during commissural neuron development.

Material and methods

Study approval

The proband and parents provided written informed consent for participation in the study, clinical data and specimen collection, genetic analysis, and publication of relevant findings under a protocol approved by the ethics committee of the Hamburg Medical Chamber (PV7038-4438-BO-ff; Hamburg, Germany).

Detailed methodologies are given in the supplemental information.

Results

We describe a 27-year-old female with mild mirror movements affecting only the hands (Table 1; see the supplemental note and Figure S1 for the full clinical description). To identify the genetic cause in the proband, we performed trio exome sequencing in the proband and healthy parents. We did not detect any rare, likely pathogenic variant in any of the known causative genes for CMMs, such as *DCC*, *NTN1*, *RAD51*, or *ARHGEF7*. However, we identified a *de novo* heterozygous 1-bp duplication, c.523dup (p.Ser175Lysfs*8), in *RBM15* (MANE Select transcript: NM_022768.5) in the proband that was confirmed by Sanger sequencing in DNA isolated from the proband's leukocytes and buccal cells (Figure 1A). The variant was absent in the proband's parents (Figure 1A). The c.523dup variant is predicted to cause a frameshift and the introduction of a premature stop codon, p.Ser175-Lysfs*8, and thus is likely a heterozygous LoF variant. To assess whether the *RBM15* variant leads to nonsense-mediated mRNA decay (NMD) of variant transcripts, we performed RT-PCR followed by Sanger sequencing using RNA (cDNA) isolated from a lymphoblastoid cell line of the proband. Both wild-type and *RBM15* transcripts containing the 1-bp duplication were detected, with sequence traces of the wild-type mRNAs approximately twice as abundant as those of the variant-containing mRNAs (Figure 1A). These findings suggest only partial NMD of the mutant *RBM15* mRNA in the proband's cells, potentially due to the variant's location within the large exon 1 (2,892 bp) of the gene.

The frameshift variant is absent in the gnomAD database (v.4.1.0)[17] and in the Regeneron Genetics Center Million Exome data.[18] *RBM15* is intolerant to LoF variants, as the LOEUF (LoF observed/expected upper bound fraction) is 0.208 and the pLI (probability of being loss-of-function intolerant) score is 1 (gnomAD v.4.1.0). Thus, *RBM15* is a highly likely haploinsufficient gene.[17] Similarly, the four autosomal-dominant disease genes for CMMs are also intolerant to LoF variants, as they have a LOEUF between 0.4 and 0.5 and, except for *ARHGEF7*, a pLI score of 1.[17] Together, based on the absence of the *RBM15* variant p.Ser175Lysfs*8 in population databases and *de novo* occurrence of the variant in the proband, the results show that the variant may underlie CMMs in the female. We received five matches on heterozygous *RBM15* variants from GeneMatcher[19]; however, the phenotypes do not fit our proband, and/or another genetic cause was identified in the affected individuals.

RBM15 is an RNA-binding protein and consists of three N-terminal RNA recognition motifs (RRMs) and a Spen paralog and ortholog C-terminal (SPOC) domain (Figure 1B). Serine 175, affected by the variant, is located in the RRM1 domain (Figure 1B). RBM15 is implicated in multiple biological processes, such as transcription, RNA modification, RNA export, X chromosome inactivation, and alternative splicing.[20] As part of a protein complex that regulates alternative splicing, RBM15 is localized in nuclear speckles, which are nuclear structures enriched in pre-mRNA splicing factors.[21] Alternative splicing of RBM15 target genes is mediated by the binding of RBM15 to specific intronic regions and recruitment of the splicing factor SF3B1 to pre-mRNA molecules.[22] The function of RBM15 in alternative splicing led us to hypothesize that this RNA-binding protein may regulate alternative splicing of *DCC* pre-mRNAs. We tested this hypothesis by carrying out an *in vitro* splicing assay. We used a previously published minigene construct that contained the genomic DNA between exons 16 and 17 of mouse *Dcc* (Figure 2A). The first 60 bp of *Dcc* exon 17 are alternatively spliced, and Nova1 and Nova2 recognize six clusters of the DNA sequence YCAY (Y = C/U) in intron 16 to promote the expression of Dcc_{long} transcripts, while the amount of Dcc_{short} mRNAs is reduced.[14] We co-transfected HEK293T cells with the *Dcc* minigene construct

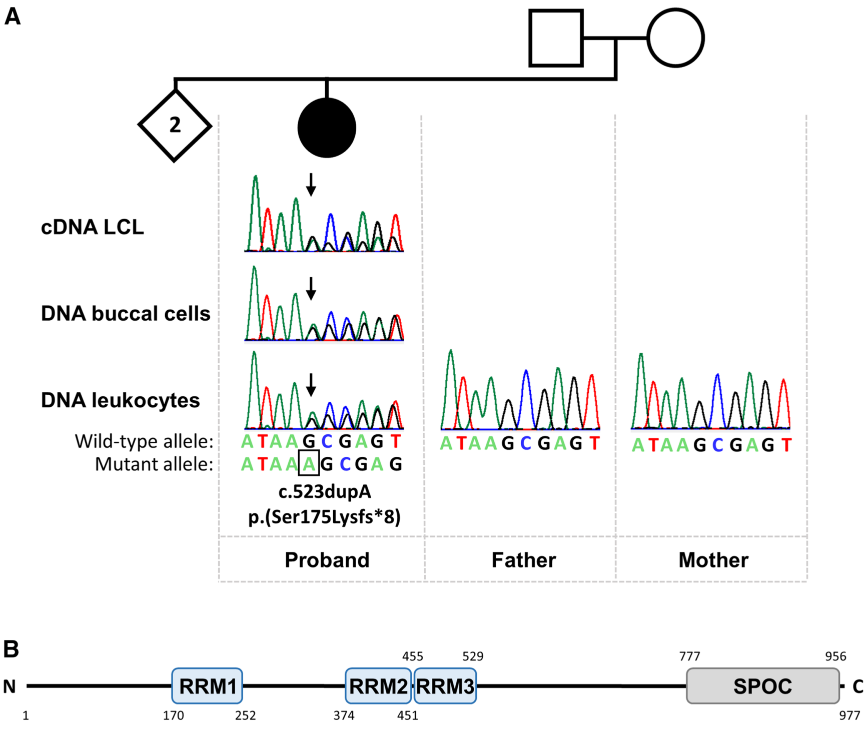

Figure 1. *De novo RBM15* frameshift variant in the proband with mirror movements, *RBM15* transcript analysis, and RBM15 domain structure

(A) The top image displays the pedigree of the proband's family. The second top image shows an electropherogram of wild-type and variant *RBM15* transcripts in cDNA derived from the proband's lymphoblastoid cell line (LCL). The middle and bottom images show partial sequence electropherograms demonstrating the heterozygous *RBM15* frameshift variant (c.523dupA [GenBank: NM_022768.5] [p.Ser175-Lysfs*8]) in genomic DNA isolated from the proband's leukocytes and buccal cells, respectively. The *RBM15* variant was absent in leukocyte-derived DNA from her healthy parents (bottom). The sequence of the wild-type and mutant alleles is given below the proband's electropherograms. The duplicated adenine is highlighted by a rectangle, and an arrow points to the start of the frameshift in the electropherograms.

(B) Domain structure of RBM15 according to UniProt database (UniProt: Q96T37). The RNA recognition motifs (RRM1–3) are represented by blue boxes and the Spen paralog and ortholog C-terminal (SPOC) domain by a gray box. Amino acid numbering is given according to the reference sequence GenBank: NP_073605.4.

and a construct expressing V5-tagged Nova1, Nova2, Rbm15, Ptbp2 (a splicing factor that does not regulate *Dcc* alternative splicing), or the empty vector (pCAGGS-V5). We confirmed expression of Nova1, Nova2, Rbm15, and Ptbp2 proteins by immunoblotting (Figure 2B). Co-expression of Nova1-V5 or Nova2-V5 with the *Dcc* minigene construct, followed by RT-PCR to amplify a *Dcc* exon 16–17 product, revealed a strong band corresponding to Dcc_{long} and a weak Dcc_{short} band (Figure 2B). Quantification of band intensities revealed proportions of Dcc_{long} and Dcc_{short} of ~76:24 and ~73:27 for Nova1 and Nova2, respectively (Figure 2C). In contrast, transfection of HEK293T cells with the empty V5 vector or the vector expressing Ptbp2-V5 resulted in similar levels of both *Dcc* transcript variants (Figure 2B), with proportions of Dcc_{long} and Dcc_{short} of ~49:51 and ~55:45 for the empty vector and Ptbp2, respectively (Figure 2C). When Rbm15 was expressed, we observed a similar effect as for Nova1 and Nova2: the band corresponding to Dcc_{long} was strong, while the one for Dcc_{short} was weak (Figure 2B). Quantitative analysis revealed proportions of Dcc_{long} and Dcc_{short} of ~69:31 (Figure 2C). Together, the data suggest that Rbm15, similar to Nova1 and Nova2, promotes inclusion of the entire exon 17 in the *Dcc* mRNA to express Dcc_{long}.

Discussion

We report here on a female with mirror movements of the hands and a heterozygous *de novo* frameshift variant, c.523dup (p.Ser175Lysfs*8), in *RBM15* that likely is a LoF allele. Transcranial magnetic stimulation results support the existence of abnormal ipsilateral corticospinal projections in addition to normally crossed corticospinal projections in the affected individual. Similarly, an abnormal uncrossed corticospinal tract has been observed in individuals with CMMs and a heterozygous *DCC*, *RAD51*, or *NTNT1* variant.[1,6,23] These data suggest that heterozygous *RBM15* LoF variants may be associated with CMMs and that RBM15 may have a function in axon guidance in the corticospinal tract.

In the spinal cord, commissural neurons extend their axons toward and across the ventral midline by integrating attractive and repulsive signals. Among the attractive cues, Netrin-1 and its receptor Dcc play key roles. Netrin-1 is an extracellular protein that binds to Dcc to guide many commissural axons within the central nervous system.[2] Depletion of Netrin-1 from the floor plate and the ventricular zone results in a failure of corticospinal tract midline crossing and leads to mirror movements in mice.[24,25] In mice mutants with a conditional deletion of *Dcc* in the corticospinal tract, however, the tract's anatomy remains normal, suggesting that Dcc may function in a non-cell-autonomous manner.[23]

Alternative splicing of *Dcc* exon 17 is mediated by Nova1 and Nova2, which promote the production of a Dcc_{long} isoform and reduce the production of Dcc_{short} to control commissural axon attraction.[14] A double *Nova1/2* knockout in the mouse spinal cord causes a failure in neuronal migration, axon outgrowth, and axon guidance

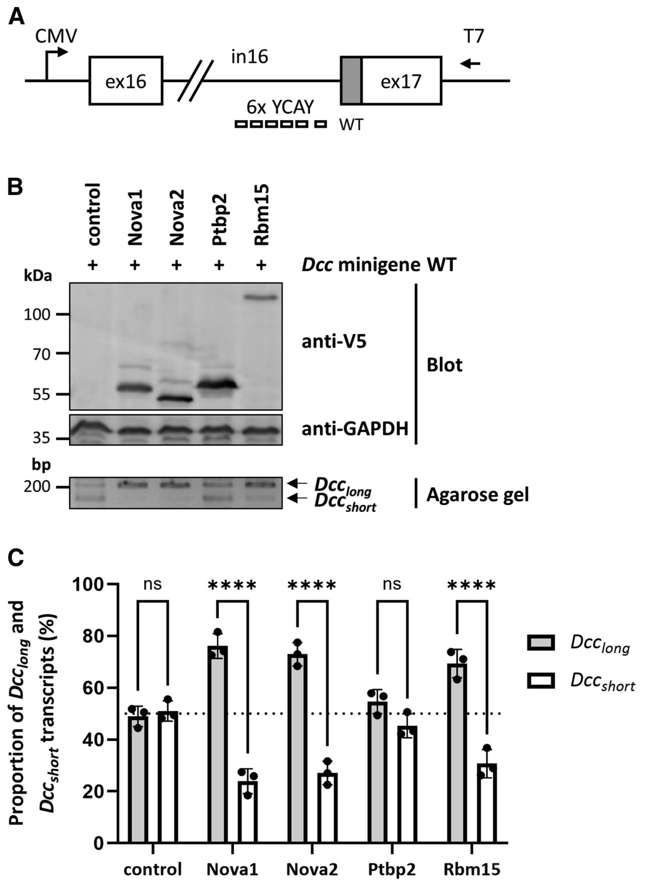

Figure 2. Rbm15 regulates alternative splicing of *Dcc* exon 17

(A) Schematics of the *Dcc* minigene construct with the genomic DNA of mouse *Dcc* exons 16 and 17 and the 5.2-kb intron 16. The CMV promoter and the position of the T7 primer binding site are indicated. The YCAY cluster (Y = C/U) composed of six YCAY repeats (rectangles) in intron 16 is shown. The alternatively spliced sequence of exon 17 (60 bp) is highlighted in gray. The figure is adapted from Figure 9 in Leggere et al.[14]

(B) HEK293T cells were transfected with the *Dcc* minigene construct together with pCAGGS plasmids expressing V5-tagged mouse Nova1, Nova2, Ptbp2, or Rbm15 or with an empty pCAGGS vector as control. After 48 h, lysates and total RNA were prepared. Expression of V5-tagged proteins was monitored with an anti-V5 antibody. An anti-GAPDH antibody was used to demonstrate equal loading. Total RNA was reverse transcribed from the T7 promoter, and semi-quantitative PCR was performed to simultaneously amplify Dcc_{long} and Dcc_{short} transcripts. A representative blot and a representative image of a 2% agarose gel are shown in the top and bottom images, respectively.

(C) Band intensities of the two PCR products were quantified by densitometric analysis. The relative abundance of Dcc_{long} and Dcc_{short} transcripts is shown, with the sum of both signal intensities set to 100%. The graph bars represent the mean ± SD of three independent experiments ($n = 3$). Individual data points and a grid line at 50% are shown. A two-way ANOVA followed by Šidák post hoc test was used for statistical analysis. ex, exon; in, intron; ns, not significant; WT, wild type; ****$p \leq 0.0001$.

of commissural interneurons, a phenotype that is similar to *Dcc* knockout. Only Dcc_{long}, not Dcc_{short}, rescues the axon projection defect in the Nova1/2 double knockout.[14] A recent study has demonstrated that the two Dcc isoforms have distinct functions, with Dcc_{short} being unable to activate Netrin-1-induced signaling, and both are required for commissural axon midline crossing. While Dcc_{long} facilitates axon growth and midline entry, Dcc_{short} reduces axon growth and allows commissural axons to exit the midline. Thus, spatially and temporally regulated expression of Dcc_{long} and Dcc_{short} during commissural neurogenesis is important for fine-tuning Netrin-1 signaling around the midline.[26] To date, RBM15-regulated alternative splicing has only been shown to be important for megakaryocyte differentiation.[22] Different conditional deletions of *Rbm15* in mice have demonstrated Rbm15's function in the development of the hematopoietic system, heart, and spleen,[27,28] while homozygous *Rbm15* germline deletion causes embryonic lethality.[28] Importantly, Rbm15 also has a role in the nervous system, such as in normal development of the mouse cerebral cortex[29] and in controlling axon outgrowth and branching and other processes to establish developmental plasticity of neurons in *Drosophila*.[30] Human *RBM15* is highly expressed in many brain regions during nervous system development, with expression levels decreasing postnatally, beginning as early as 4 months of age. However, relatively high *RBM15* expression persists in the cerebellar cortex until 30–37 years of age (Figure S2). Based on these data, it will be important to study Rbm15's function in the corticospinal tract and whether Rbm15 is required for migration, axon outgrowth, and/or guidance of commissural neurons. For example, *Rbm15* knockout or knockdown studies in the spinal cord using whole mouse embryo culture are required to show a potential role of Rbm15 in midline crossing of axons and in the regulation of *Dcc* alternative splicing. Moreover, the identification of additional individuals with CMMs and heterozygous *RBM15* variants is important to provide further evidence for the provisional Mendelian gene discovery reported here.

Data and code availability

The published article includes all data generated or analyzed during this study. Consent restrictions preclude sharing of full data sets, and the consents do not cover the deposition of the exome sequencing data in a public database. *RBM15* variant and phenotypic information were submitted to the LOVD database (https://databases.lovd.nl/shared/genes/RBM15), with the LOVD Variant ID #0001030018.

Acknowledgments

We are grateful to the proband and parents who agreed to participate in this project. We thank Lara Adrian and Henrike Wilshusen for skillful technical assistance. This work was supported by the Deutsche Forschungsgemeinschaft (KU 1240/17-1 to K.K.).

Declaration of interests

The authors declare no competing interests.

Web resources

GenBank, https://www.ncbi.nlm.nih.gov/genbank/
GeneMatcher, https://genematcher.org/
gnomAD, https://gnomad.broadinstitute.org
Human Phenotype Ontology (HPO), https://hpo.jax.org/
Online Mendelian Inheritance in Man (MIM), http://www.omim.org/
Regeneron Genetics Center Million Exome Variant Browser, https://rgc-research.regeneron.com/me/home
UniProt, https://www.uniprot.org/

Supplemental information

Supplemental information can be found online at https://doi.org/10.1016/j.xhgg.2025.100528.

Received: April 28, 2025
Accepted: October 3, 2025

References

1. Gallea, C., Popa, T., Hubsch, C., Valabregue, R., Brochard, V., Kundu, P., Schmitt, B., Bardinet, E., Bertasi, E., Flamand-Roze, C., et al. (2013). RAD51 deficiency disrupts the corticospinal lateralization of motor control. Brain *136*, 3333–3346. https://doi.org/10.1093/brain/awt258.
2. Roze, E., Dubacq, C., and Welniarz, Q. (2025). Corticospinal Tract Development, Evolution, and Skilled Movements. Mov. Disord. *40*, 1221–1232. https://doi.org/10.1002/mds.30199.
3. Welniarz, Q., Gallea, C., Lamy, J.C., Méneret, A., Popa, T., Valabregue, R., Béranger, B., Brochard, V., Flamand-Roze, C., Trouillard, O., et al. (2019). The supplementary motor area modulates interhemispheric interactions during movement preparation. Hum. Brain Mapp. *40*, 2125–2142. https://doi.org/10.1002/hbm.24512.
4. Srour, M., Rivière, J.B., Pham, J.M.T., Dubé, M.P., Girard, S., Morin, S., Dion, P.A., Asselin, G., Rochefort, D., Hince, P., et al. (2010). Mutations in DCC cause congenital mirror movements. Science *328*, 592. https://doi.org/10.1126/science.1186463.
5. Depienne, C., Bouteiller, D., Méneret, A., Billot, S., Groppa, S., Klebe, S., Charbonnier-Beaupel, F., Corvol, J.C., Saraiva, J.P., Brueggemann, N., et al. (2012). RAD51 haploinsufficiency causes congenital mirror movements in humans. Am. J. Hum. Genet. *90*, 301–307. https://doi.org/10.1016/j.ajhg.2011.12.002.
6. Meneret, A., Franz, E.A., Trouillard, O., Oliver, T.C., Zagar, Y., Robertson, S.P., Welniarz, Q., Gardner, R.J.M., Gallea, C., Srour, M., et al. (2017). Mutations in the netrin-1 gene cause congenital mirror movements. J. Clin. Investig. *127*, 3923–3936. https://doi.org/10.1172/JCI95442.
7. Schlienger, S., Yam, P.T., Balekoglu, N., Ducuing, H., Michaud, J.F., Makihara, S., Kramer, D.K., Chen, B., Fasano, A., Berardelli, A., et al. (2023). Genetics of mirror movements identifies a multifunctional complex required for Netrin-1 guidance and lateralization of motor control. Sci. Adv. *9*, eadd5501. https://doi.org/10.1126/sciadv.add5501.
8. Ahmed, I., Mittal, K., Sheikh, T.I., Vasli, N., Rafiq, M.A., Mikhailov, A., Ohadi, M., Mahmood, H., Rouleau, G.A., Bhatti, A., et al. (2014). Identification of a homozygous splice site mutation in the dynein axonemal light chain 4 gene on 22q13.1 in a large consanguineous family from Pakistan with congenital mirror movement disorder. Hum. Genet. *133*, 1419–1429. https://doi.org/10.1007/s00439-014-1475-8.
9. Collins Hutchinson, M.L., St-Onge, J., Schlienger, S., Boudrahem-Addour, N., Mougharbel, L., Michaud, J.F., Lloyd, C., Bruneau, E., Roux, C., Sahly, A.N., et al. (2024). Defining the Genetic Landscape of Congenital Mirror Movements in 80 Affected Individuals. Mov. Disord. *39*, 400–410. https://doi.org/10.1002/mds.29669.
10. Franz, E.A., Chiaroni-Clarke, R., Woodrow, S., Glendining, K. A., Jasoni, C.L., Robertson, S.P., Gardner, R.J.M., and Markie, D. (2015). Congenital mirror movements: phenotypes associated with DCC and RAD51 mutations. J. Neurol. Sci. *351*, 140–145. https://doi.org/10.1016/j.jns.2015.03.006.
11. Meneret, A., Depienne, C., Riant, F., Trouillard, O., Bouteiller, D., Cincotta, M., Bitoun, P., Wickert, J., Lagroua, I., Westenberger, A., et al. (2014). Congenital mirror movements: mutational analysis of RAD51 and DCC in 26 cases. Neurology *82*, 1999–2002. https://doi.org/10.1212/WNL.0000000000000477.
12. Keino-Masu, K., Masu, M., Hinck, L., Leonardo, E.D., Chan, S. S., Culotti, J.G., and Tessier-Lavigne, M. (1996). Deleted in Colorectal Cancer (DCC) encodes a netrin receptor. Cell *87*, 175–185. https://doi.org/10.1016/s0092-8674(00)81336-7.
13. Kennedy, T.E., Serafini, T., de la Torre, J.R., and Tessier-Lavigne, M. (1994). Netrins are diffusible chemotropic factors for commissural axons in the embryonic spinal cord. Cell *78*, 425–435. https://doi.org/10.1016/0092-8674(94)90421-9.
14. Leggere, J.C., Saito, Y., Darnell, R.B., Tessier-Lavigne, M., Junge, H.J., and Chen, Z. (2016). NOVA regulates Dcc alternative splicing during neuronal migration and axon guidance in the spinal cord. eLife *5*, e14264. https://doi.org/10.7554/eLife.14264.
15. Reale, M.A., Hu, G., Zafar, A.I., Getzenberg, R.H., Levine, S. M., and Fearon, E.R. (1994). Expression and alternative splicing of the deleted in colorectal cancer (DCC) gene in normal and malignant tissues. Cancer Res. *54*, 4493–4501.
16. Saito, Y., Miranda-Rottmann, S., Ruggiu, M., Park, C.Y., Fak, J.J., Zhong, R., Duncan, J.S., Fabella, B.A., Junge, H.J., Chen, Z., et al. (2016). NOVA2-mediated RNA regulation is required for axonal pathfinding during development. eLife *5*, e14371. https://doi.org/10.7554/eLife.14371.
17. Chen, S., Francioli, L.C., Goodrich, J.K., Collins, R.L., Kanai, M., Wang, Q., Alföldi, J., Watts, N.A., Vittal, C., Gauthier, L.D., et al. (2024). A genomic mutational constraint map using variation in 76,156 human genomes. Nature *625*, 92–100. https://doi.org/10.1038/s41586-023-06045-0.
18. Sun, K.Y., Bai, X., Chen, S., Bao, S., Zhang, C., Kapoor, M., Backman, J., Joseph, T., Maxwell, E., Mitra, G., et al. (2024). A deep catalogue of protein-coding variation in 983,578 individuals. Nature *631*, 583–592. https://doi.org/10.1038/s41586-024-07556-0.
19. Sobreira, N., Schiettecatte, F., Boehm, C., Valle, D., and Hamosh, A. (2015). New tools for Mendelian disease gene identification: PhenoDB variant analysis module; and GeneMatcher, a web-based tool for linking investigators with an interest in the same gene. Hum. Mutat. *36*, 425–431. https://doi.org/10.1002/humu.22769.
20. Cao, Y., Qiu, G., Dong, Y., Zhao, W., and Wang, Y. (2024). Exploring the role of m (6) A writer RBM15 in cancer: a systematic review. Front. Oncol. *14*, 1375942. https://doi.org/10.3389/fonc.2024.1375942.

21. Horiuchi, K., Kawamura, T., Iwanari, H., Ohashi, R., Naito, M., Kodama, T., and Hamakubo, T. (2013). Identification of Wilms' tumor 1-associating protein complex and its role in alternative splicing and the cell cycle. J. Biol. Chem. *288*, 33292–33302. https://doi.org/10.1074/jbc.M113.500397.
22. Zhang, L., Tran, N.T., Su, H., Wang, R., Lu, Y., Tang, H., Aoyagi, S., Guo, A., Khodadadi-Jamayran, A., Zhou, D., et al. (2015). Cross-talk between PRMT1-mediated methylation and ubiquitylation on RBM15 controls RNA splicing. eLife *4*, e07938. https://doi.org/10.7554/eLife.07938.
23. Welniarz, Q., Morel, M.P., Pourchet, O., Gallea, C., Lamy, J. C., Cincotta, M., Doulazmi, M., Belle, M., Méneret, A., Trouillard, O., et al. (2017). Non cell-autonomous role of DCC in the guidance of the corticospinal tract at the midline. Sci. Rep. *7*, 410. https://doi.org/10.1038/s41598-017-00514-z.
24. Hu, L., Liu, X.Y., Zhao, L., Hu, Z.B., Li, Z.X., Liu, W.T., Song, N.N., Hu, Y.Q., Jiang, L.P., Zhang, L., et al. (2024). Ventricular Netrin-1 deficiency leads to defective pyramidal decussation and mirror movement in mice. Cell Death Dis. *15*, 343. https://doi.org/10.1038/s41419-024-06719-1.
25. Pourchet, O., Morel, M.P., Welniarz, Q., Sarrazin, N., Marti, F., Heck, N., Galléa, C., Doulazmi, M., Roig Puiggros, S., Moreno-Bravo, J.A., et al. (2021). Loss of floor plate Netrin-1 impairs midline crossing of corticospinal axons and leads to mirror movements. Cell Rep. *34*, 108654. https://doi.org/10.1016/j.celrep.2020.108654.
26. Dailey-Krempel, B., Martin, A.L., Jo, H.N., Junge, H.J., and Chen, Z. (2023). A tug of war between DCC and ROBO1 signaling during commissural axon guidance. Cell Rep. *42*, 112455. https://doi.org/10.1016/j.celrep.2023.112455.
27. Raffel, G.D., Chu, G.C., Jesneck, J.L., Cullen, D.E., Bronson, R.T., Bernard, O.A., and Gilliland, D.G. (2009). Ott1 (Rbm15) is essential for placental vascular branching morphogenesis and embryonic development of the heart and spleen. Mol. Cell Biol. *29*, 333–341. https://doi.org/10.1128/MCB.00370-08.
28. Raffel, G.D., Mercher, T., Shigematsu, H., Williams, I.R., Cullen, D.E., Akashi, K., Bernard, O.A., and Gilliland, D.G. (2007). Ott1(Rbm15) has pleiotropic roles in hematopoietic development. Proc. Natl. Acad. Sci. USA *104*, 6001–6006. https://doi.org/10.1073/pnas.0609041104.
29. Xie, Y., Castro-Hernández, R., Sokpor, G., Pham, L., Narayanan, R., Rosenbusch, J., Staiger, J.F., and Tuoc, T. (2019). RBM15 Modulates the Function of Chromatin Remodeling Factor BAF155 Through RNA Methylation in Developing Cortex. Mol. Neurobiol. *56*, 7305–7320. https://doi.org/10.1007/s12035-019-1595-1.
30. Gu, T., Zhao, T., Kohli, U., and Hewes, R.S. (2017). The large and small SPEN family proteins stimulate axon outgrowth during neurosecretory cell remodeling in Drosophila. Dev. Biol. *431*, 226–238. https://doi.org/10.1016/j.ydbio.2017.09.013.

HGGA, Volume *7*

Supplemental information

A *de novo* frameshift variant in the candidate *RBM15* in a proband with congenital mirror movements

Frederike L. Harms, Fanny Kortüm, Malik Alawi, Martin Staudt, and Kerstin Kutsche

Supplemental Information

Supplemental case report

We describe a 27-year-old female with mirror movements. The proband is the third child of healthy non-consanguineous parents. She has two elder siblings, one is healthy, while the other one was diagnosed with multiple sclerosis. The proband's pregnancy and birth were uneventful. Motor development was slightly delayed (independent walking at >18 months of age), other milestones were reached at appropriate ages. At the age of 3-4 years, the parents noticed their daughter's pronounced mirror movements while drawing.

At the age of 7 years, she was referred to M.S. (Department of Pediatric Neurology and Developmental Medicine, University Children's Hospital Tübingen, Germany) for the evaluation of mirror movements. Her parents had noticed that during intentional movements of one hand, the other hand performed involuntary movements "mirroring" the intended movements in the task hand. These mirror movements could be suppressed, at least to some degree, by voluntarily increasing the muscle tone in the non-task hand, but complete suppression was not possible. Apart from mirror movements, standard neurological examination was normal, and cranial MRI was also normal. Transcranial magnetic stimulation (TMS; MagStim 200; focal figure-eight-coil; bilateral registration of motor evoked potentials from M. interosseus dorsalis I via surface EMG electrodes), however, demonstrated abnormalities in the organization of the corticospinal tract, with prominent fast-conducting ipsilateral projections: TMS of the right hemisphere elicited motor evoked potentials (MEPs) in both hands (80% stimulator output; no pre-contraction), with latencies of 17.1 ms (left hand) and 17.7 ms (right hand). Similarly, TMS of the left hemisphere (100% stimulator output) also elicited MEPs in both hands (no pre-contraction), with latencies of 17.4 ms (right hand) and 18.3 ms (left hand).

At the age of 20 years, she was again evaluated by M.S. (Center for Pediatric Neurology and Neurorehabilitation, Schön Clinic Vogtareuth, Germany). The proband reported only minimal problems during activities of daily living: after long periods of writing or drawing by hand (with her right hand), the muscle tone in her (non-writing) left hand increased slowly and sometimes caused minor pain. The

proband used to play the trumpet for several years, but had stopped playing. She tried playing the piano, but it was not possible. She was a good volleyball-player for a period of 6-7 years. In school, she described herself as talented in languages, but rather weak in mathematics, where she had required additional support to obtain the secondary school certificate. Neurological examination revealed marked mirror movements during some activities (e.g. untying shoelaces), which were less marked but still present in the artificial situation of a neurological examination, e.g. during flexing / extending fingers and pro- / supination. No mirror movements were observed in the more proximal joints of the upper extremity and in the lower extremity. When nervous, the proband showed minimal intention tremor (left > right), brisk deep tendon reflexes, but negative pyramidal tract signs. TMS of the right hemisphere elicited MEPs not only in the contralateral left hand (resting motor threshold (RMT): 33%, latency at 110% RMT: 21.0 ms), but also in the right hand (RMT 50%, latency at 110% RMT: 20.6 ms). TMS of the left hemisphere elicited MEPs not only in the contralateral right hand (resting motor threshold (RMT): 36%, latency at 110% RMT: 21.2 ms), but also in the left hand (RMT 42%, latency at 110% RMT: 22.2 ms). Thus, TMS examinations at 7 and 20 years of age demonstrated that both hemispheres of the proband had normally crossed corticospinal projections and abnormal ipsilateral corticospinal projections (requiring somewhat higher stimulation intensities than for the crossed projections – this could be demonstrated in the second TMS study, where a more cooperative proband allowed a more detailed examination).

At the age of 26 years, the proband experienced sensory disturbances affecting the left arm and the left side of the face, which prompted her hospital admission. Examinations excluded intracranial and spinal causes, as well as inflammatory central nervous system diseases, as the underlying source of her symptoms. Brain MRI revealed unremarkable infratentorial and supratentorial findings (**Supplemental Figure 1**).

In a telephone interview, the 27-year-old proband reported no current limitations in her daily living (except for the inability to play the piano). She had continued her school career and obtained the advanced technical college certificate, went to college and graduated as a specialist for media and

information technology. In her job she works in an office and uses her own “6-finger typing system”.

She had tried to learn the 10-finger typing system, but has not been successful.

Supplemental figures and legends

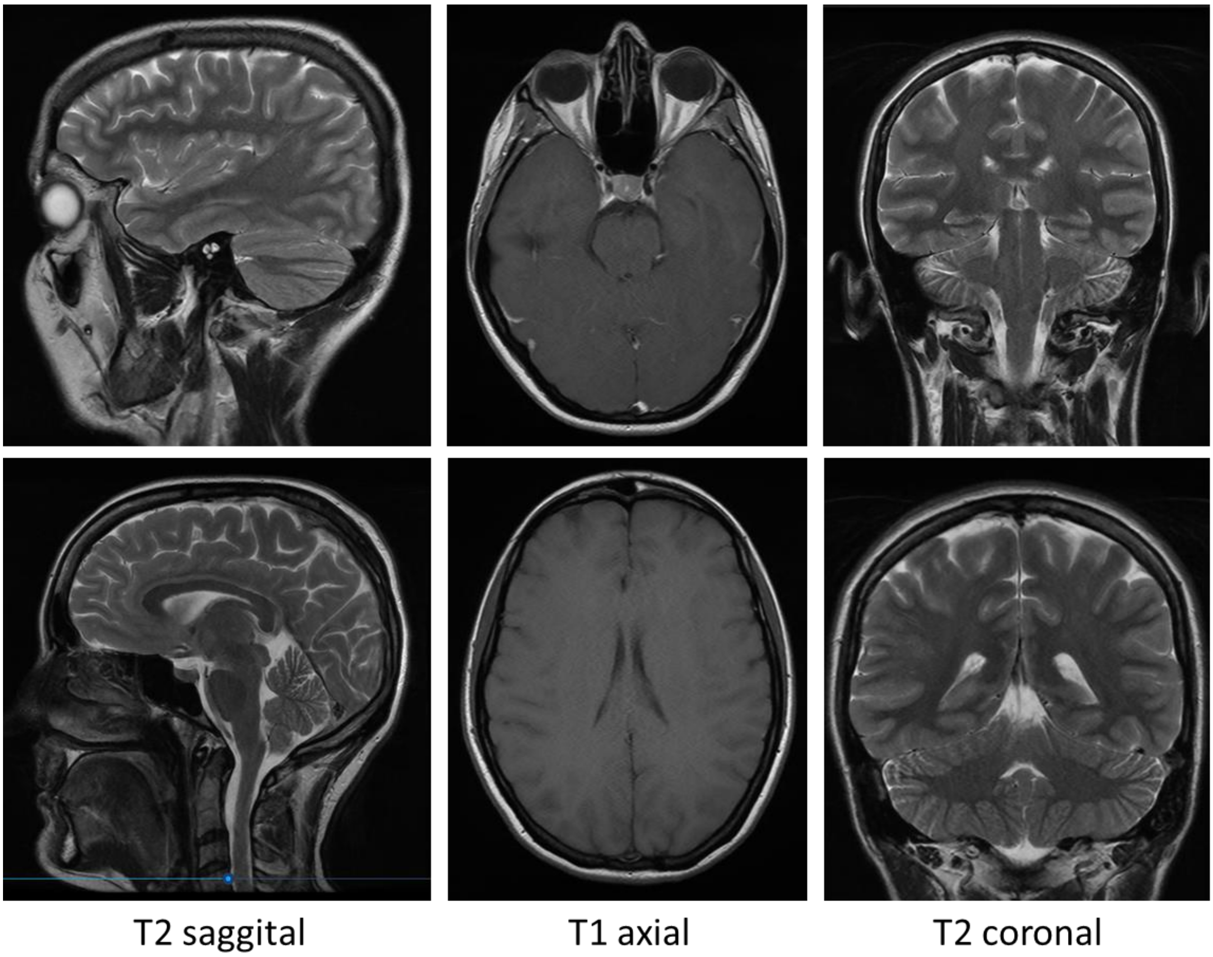

Supplemental Figure 1. Selected brain MRI scans of the proband at the age of 26 years

Sagittal (left) and coronal (right) T2-weighted images, as well as axial (middle) T1-weighted images, show unremarkable infratentorial and supratentorial findings.

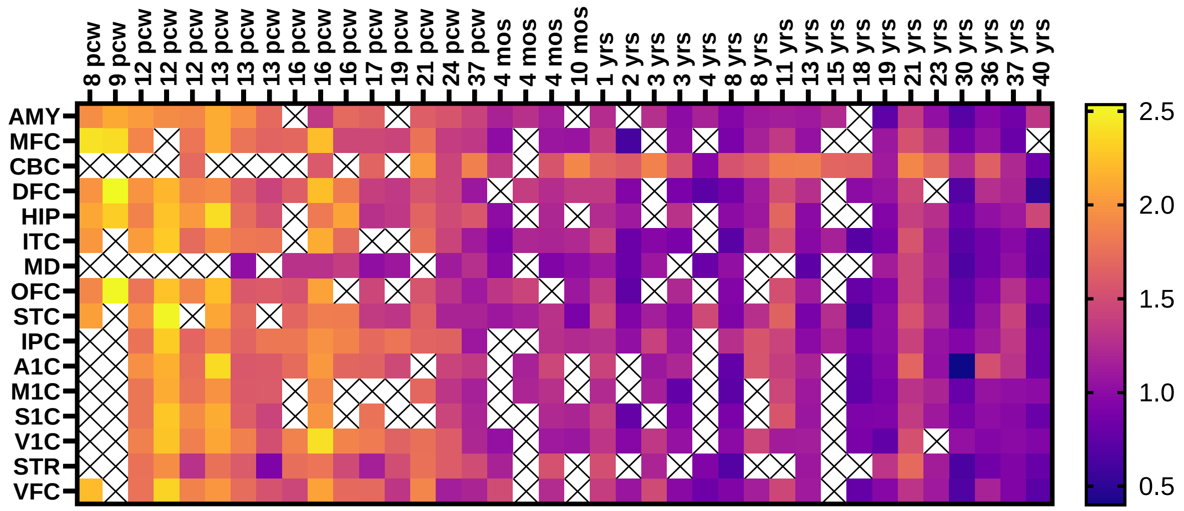

Supplemental Figure 2. Spatio-temporal expression of *RBM15* mRNA in the human brain

The heatmap illustrates relative *RBM15* mRNA expression across human brain samples from donors aged 8 to 37 post-conception weeks (pcw) and from 4 months (mos) to 40 years (yrs). Expression was analyzed in the following brain regions: amygdaloid complex (AMY), anterior (rostral) cingulate (medial prefrontal) cortex (MFC), cerebellar cortex (CBC), dorsolateral prefrontal cortex (DFC), hippocampus (HIP), inferolateral temporal cortex (ITC), mediodorsal thalamic nucleus (MD), orbital frontal cortex (OFC), posterior (caudal) superior temporal cortex (STC), posteroventral (inferior) parietal cortex (IPC), primary auditory cortex (A1C), primary motor cortex (M1C), primary somatosensory cortex (S1C), primary visual cortex (V1C), striatum (STR), and ventrolateral prefrontal cortex (VFC). Dark violet indicates low expression and yellow high expression (log_2 RPKM (reads per kilobase of transcript); scale shown on the right). Data were obtained from the *BrainSpan Atlas of the Developing Human Brain* (© 2016 Allen Institute for Brain Science. Aging, Dementia and TBI study; available at: https://www.brainspan.org/).[4]

Supplemental Table

Supplemental Table 1. Sequence of oligonucleotides used in this work

***RBM15* primer sequences for variant validation**			
**Template**	**Exon**	**Direction**	**Sequence (5' → 3')**
DNA	1	forward	GTG GGG GCA GCT CCA GTA
		reverse	AGA GGC CGG TCA TAG AGC AC
***RBM15* primer sequences for transcript analysis (RT-PCR)**			
**Template**	**Exon**	**Direction**	**Sequence (5' → 3')**
cDNA	1	forward	GCT CCA GTA GCC GCT TGC
	2	reverse	AAC TCC TAT CTG AAA ACC AAA CC
**Primer sequences for cloning the mouse *Rbm15* coding region into pCAGGS-MCS-V5**			
**Template**		**Direction**	**Sequence (5' → 3')**
cDNA from mouse brain		forward	TTG GCA AAG AAT TCG GTA CCA TGA GGT CTG CGG GGC GG
		reverse	GTG CTC GAG GCG GCC TCC GCT GTT CAC CAG TTT TGC
**Primer sequences for *Dcc* minigene assay**			
**Task**		**Direction**	**Sequence (5' → 3')**
Reverse transcription from T7 promotor		reverse	CTA TAG TGA GTC GTA TTA
Amplification of Dcc_{long} and Dcc_{short} transcripts		forward	TCT CAT TAT GTA ATC TCC TTA AAA GC
		reverse	TCA CAG CCT CAT GGG TAA GAG

Supplemental methods

Exome sequencing

DNA was isolated by standard procedures. Trio exome sequencing (ES) was performed on leukocyte-derived DNA from the proband and her parents as described previously.[1] Briefly, enrichment was carried out using the Nextera Enrichment kit (62 Mb) (Illumina). Captured libraries were then loaded and sequenced on the HiSeq2000 platform (Illumina, San Diego, CA). Trimmomatic was employed to remove adapters, low quality (phred quality score < 5) bases from the 3' ends of sequence reads.[2] Reads shorter than 36 bp were subsequently removed. Further processing was performed following the Genome Analysis Toolkit's (GATK) best practice recommendations. Briefly, trimmed reads were aligned to the human reference genome (UCSC GRCh37/hg19) using the Burrows-Wheeler Aligner (BWA mem v0.7.12). Duplicate reads were marked with Picard tools (v1.141). GATK (v3.4) was employed for indel realignment, base quality score recalibration, calling variants using the HaplotypeCaller, joint genotyping, and variant quality score recalibration. AnnoVar (v2015-03-22) was used to functionally annotate and filter alterations against public databases (dbSNP138, 1000 Genomes Project, and ExAC Browser). Only exonic and intronic variants that were *de novo* (absent from public databases) or rare (with a minor allele frequency [MAF] ≤0.1% and no homo- and hemizygotes in public databases) were retained. Variants with poor depth of sequencing coverage (total read depth < 10) and in low quality regions (checked in IGV) were discarded.

Variant validation

RBM15 variant validation was performed by Sanger-sequencing using leukocyte-derived DNA from the proband and parents and DNA isolated from buccal cells of the proband. Primers designed to amplify the selected region of *RBM15* exon 1 (NM_022768.5) are described in **Supplemental Table 1**. Amplicons were directly sequenced using the ABI BigDye Terminator Sequencing Kit (Applied Biosystems) and an automated capillary sequencer (ABI 3500, Applied Biosystems). Sequence electropherograms were analyzed using Chromas v2.6.6 (Technelysium Pty Ltd).

Plasmid information and cloning procedures

pCAGGS-Nova1-V5, pCAGGS-Nova2-V5, pCAGGS-Ptbp2-V5, pCAGGS-V5 empty vector, and wild-type and mutant *Dcc* minigene constructs (1xYCAY mut, 4xYCAY mut, 5xYCAY mut, and 6xYCAY mut) in pDEST26 backbones were kindly provided by Harald J. Junge (Department of Ophthalmology and Visual Neurosciences, University of Minnesota, Minneapolis, MN 55455, USA) and Zhe Chen (Department of Neuroscience, University of Minnesota, Minneapolis, MN 55455, USA).[3]

To generate a construct for expression of C-terminally V5-tagged Rbm15 (pCAGGS-Rbm15-V5), we amplified the coding region of mouse *Rbm15* (NM_001045807.2) using primers and cDNA of mouse brain as a template. The purified PCR product was then cloned between the *Kpn*I and *Not*I restriction sites of pCAGGS-V5 using the InFusion HD Cloning Kit (Takara). All constructs were regularly sequenced for integrity and primer sequences for InFusion cloning are described in **Supplemental Table 1**.

Cell culture conditions

HEK293T were cultured in Dulbecco's modified Eagle medium (DMEM; Thermo Fisher Scientific) supplemented with 10% fetal bovine serum (FBS; GE Healthcare) and penicillin-streptomycin (100 U/mL and 100 mg/mL, respectively; Thermo Fisher Scientific). The proband-derived lymphoblastoid cell line was maintained in RPMI 1640 medium (Gibco) supplemented with 20% FBS and penicillin-streptomycin.

***RBM15* transcript analysis**

Total RNA was extracted from proband-derived lymphoblastoid cells using the RNeasy Mini Kit (Qiagen). 1 µg total RNA was reverse transcribed using oligo(dT) primer, and semi-quantitative RT-PCR was performed to amplify *RBM15* transcripts (primer sequences are listed in **Supplemental Table 1**). The resulting RT-PCR products were directly Sanger sequenced using the forward primer.

Dcc minigene assay

The *Dcc* minigene assay was adapted from Leggere *et al.* (2016).[3] Briefly, HEK293T cells were transiently transfected with wild-type or mutant *Dcc* minigene construct together with pCAGGS-Nova1-V5, pCAGGS-Nova2-V5, pCAGGS-Ptbp2-V5, pCAGGS-Rbm15-V5, or pCAGGS-V5 empty vector using TurboFect transfection reagent (Thermo Fisher Scientific) following the manufacturer's protocol. Transfection medium was changed 4-6 hours after transfection and cells were cultured in 10% DMEM for 48 h. Subsequently, total RNA was extracted using RNeasy Mini Kit (Qiagen) and whole-cell lysates were collected in ice-cold RIPA buffer [50 mM Tris-HCl, pH 8.0; 150 mM NaCl; 1% NP-40; 0.5% DOC (sodium deoxycholate); 0.1% SDS (sodium dodecyl sulfate)] supplemented with Mini Protease Inhibitor (Roche).

The concentration and purity of the RNA samples were assessed using the Epoch™ Microplate Spectrophotometer (BioTek). 1 µg total RNA was reverse transcribed from the T7 promoter and semi-quantitative PCR was performed to amplify Dcc_{long} and Dcc_{short} transcripts (for primer sequences see **Supplemental Table 1**). For analysis, PCR products were separated on a 2% agarose gel and band intensities were quantified by densitometric analysis using the ImageJ software.

Expression of V5-tagged proteins in whole-cell lysates was confirmed with immunoblotting using mouse monoclonal anti-V5 (Invitrogen; #R960-25; clone SV5-Pk1; 1:5,000 dilution) as primary and horseradish peroxidase-conjugated sheep anti-mouse (GE Healthcare; NA931V; 1:10,000 dilution) as secondary antibody. For control of equal loading, whole-cell lysates were analyzed using a mouse monoclonal anti-GAPDH antibody (Abcam; #ab8245; 1:10,000 dilution). Immunoblots were digitally imaged using a ChemiDoc system (Bio-Rad), with exposure time optimized to avoid saturation.

Data analysis and statistics

Quantitative data are presented by GraphPad Prism 8 software (Instat, GraphPad Software) as the mean ± standard deviation (SD). For quantification, two-way ANOVA followed by a Šidák *post hoc* test for multiple comparisons was performed. A *p*-value ≤0.05 was considered statistically significant.

Supplemental references

1. Kortum, F., Caputo, V., Bauer, C.K., Stella, L., Ciolfi, A., Alawi, M., Bocchinfuso, G., Flex, E., Paolacci, S., Dentici, M.L., et al. (2015). Mutations in KCNH1 and ATP6V1B2 cause Zimmermann-Laband syndrome. Nat Genet *47*, 661-667. 10.1038/ng.3282.
2. Bolger, A.M., Lohse, M., and Usadel, B. (2014). Trimmomatic: a flexible trimmer for Illumina sequence data. Bioinformatics *30*, 2114-2120. 10.1093/bioinformatics/btu170.
3. Leggere, J.C., Saito, Y., Darnell, R.B., Tessier-Lavigne, M., Junge, H.J., and Chen, Z. (2016). NOVA regulates Dcc alternative splicing during neuronal migration and axon guidance in the spinal cord. Elife *5*, e14264. 10.7554/eLife.14264.
4. Miller, J.A., Guillozet-Bongaarts, A., Gibbons, L.E., Postupna, N., Renz, A., Beller, A.E., Sunkin, S.M., Ng, L., Rose, S.E., Smith, K.A., et al. (2017). Neuropathological and transcriptomic characteristics of the aged brain. Elife *6,* e31126. 10.7554/eLife.31126.
